# 7-Chloro-2-[1-(4-methoxy­phen­yl)pyrazol-4-yl]-3,3-dimethyl-3*H*-indole

**DOI:** 10.1107/S1600536809052581

**Published:** 2009-12-12

**Authors:** Madeleine Helliwell, Arash Afghan, Foroogh Keshvari, Mehdi M. Baradarani, John A. Joule

**Affiliations:** a The School of Chemistry, The University of Manchester, Manchester M13 9PL, England; b Faculty of Petroleum Chemistry, Urmia University of Technology, Urmia, Iran; c Department of Chemistry, Faculty of Science, University of Urmia, Urmia 57135, Iran

## Abstract

In the title compound, C_20_H_18_ClN_3_O, the dihedral angle between the pyrazole and the 3*H*-indole components is only 13.28 (6)°, indicating that there is conjugation between the two heterocyclic subunits. The *N*-methoxy­phenyl unit makes a dihedral angle of 25.10 (7)° with the pyrazole ring.

## Related literature

For related structures, see: Baradarani *et al.* (2006 ▶); Rashidi *et al.* (2009 ▶).
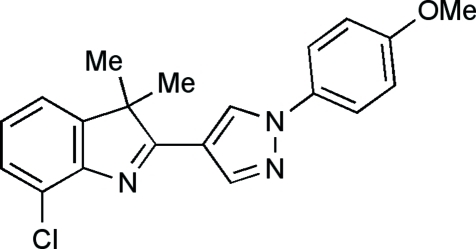


## Experimental

### 

#### Crystal data

C_20_H_18_ClN_3_O
*M* *_r_* = 351.82
Monoclinic, 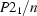

*a* = 11.635 (3) Å
*b* = 10.328 (3) Å
*c* = 14.141 (4) Å
β = 95.681 (5)°
*V* = 1690.9 (8) Å^3^
*Z* = 4
Mo *K*α radiation
μ = 0.24 mm^−1^
*T* = 100 K
0.50 × 0.40 × 0.20 mm

#### Data collection

Bruker SMART APEX CCD area-detector diffractometer
9553 measured reflections
3461 independent reflections
2662 reflections with *I* > 2σ(*I*)
*R*_int_ = 0.092

#### Refinement

*R*[*F* ^2^ > 2σ(*F* ^2^)] = 0.049
*wR*(*F* ^2^) = 0.124
*S* = 0.97
3461 reflections
229 parameters
H-atom parameters constrained
Δρ_max_ = 0.70 e Å^−3^
Δρ_min_ = −0.30 e Å^−3^

### 

Data collection: *SMART* (Bruker, 2001 ▶); cell refinement: *SAINT* (Bruker, 2002 ▶); data reduction: *SAINT*; program(s) used to solve structure: *SHELXS97* (Sheldrick, 2008 ▶); program(s) used to refine structure: *SHELXL97* (Sheldrick, 2008 ▶); molecular graphics: *SHELXTL* (Sheldrick, 2008 ▶); software used to prepare material for publication: *SHELXTL*.

## Supplementary Material

Crystal structure: contains datablocks global, I. DOI: 10.1107/S1600536809052581/ez2195sup1.cif

Structure factors: contains datablocks I. DOI: 10.1107/S1600536809052581/ez2195Isup2.hkl

Additional supplementary materials:  crystallographic information; 3D view; checkCIF report

## Figures and Tables

**Fig. 1.**
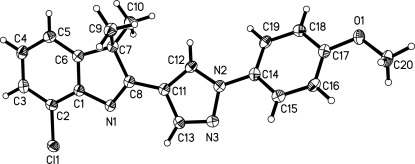
View of the title compound showing the atom numbering scheme with 50% probability displacement ellipsoids.

**Fig. 2.**
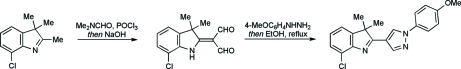
The formation of the title compound.

## supplementary crystallographic information

### Comment

We have been studying the interaction of 2,3,3-trimethyl-3*H*-indoles with the Vilsmeier reagent and discovered that this produced (3,3-dimethyl-2,3-dihydroindol-2-ylidene)malondialdehydes (Baradarani *et al.*, 2006). 2,3,3-trimethyl-3*H*-pyrrolo[2,3-*f*]quinoline and 2,3,3-trimethyl-3*H*-pyrrolo[3,2-*h*]quinoline behave similarly (Rashidi *et al.*, 2009). The malondialdehydes could be reacted in turn with arylhydrazines to produce mono-arylhydrazones which, on simple reflux in ethanol, were converted into 3,3-dimethyl-2-[1-aryl-1*H* -pyrazol-4-yl]-3*H*-indoles (Scheme 2). We now report the crystallographically determined structure of one of these: (1-(4-methoxyphenyl)pyrazol-4-yl)-3*H*-indole (1).

The structure of (1) reveals the extent of conjugation of the two heterocyclic components of the molecule, *i.e.* the pyrazole and the 3*H*-indole. Each of these two components is essential planar; the greatest distance from the least squares plane through the atoms C11 - C13, N2, N3 is 0.004 (2) Å for C11 and the greatest distance from the least squares plane through the atoms C1—C8, N1 is 0.027 (2) Å for C7. Furthermore, the dihedral angle between these two planes is only 13.28 (6) °, indicating conjugation between the two aromatic heterocycles. The *N*-methoxyphenyl unit, perhaps surprisingly, is not coplanar with its attached pyrazole making a dihedral angle of 25.10 (7) ° with the pyrazole unit.

### Experimental

A mixture of 7-chloro-3,3-dimethyl-2,3-dihydroindol- 2-ylidene)malondialdehyde (150 mg, 0.6 mmol) and 4-methoxyphenylhydrazine hydrochloride (110 mg, 0.6 mmol) in ethanol (15 ml) was heated at reflux for 2 h. After this time, the solvent was evaporated and residue recrystallized from absolute ethanol to give 7-chloro-3,3-dimethyl-2- (1-(4-methoxyphenyl)pyrazol-4-yl)-3*H*-indole (179 mg, 85%). m.p. 464–465 K. ^1^H-NMR (CDCl_3_) δ 1.56 (6*H*, s, 2xMe), 3.88 (3*H*, s, OMe), 7.02 (2*H*, d, *J* = 9 Hz, Ar—H), 7.17 (t, *J* = 7.5 Hz, 1H, H-5), 7.24 (dd, *J* = 7.5, 1.2 Hz, H-4), 7.35 (1*H*, dd, *J* = 7.5, 1.2 Hz, H-6), 7.69 (2*H*, d, *J* = 9 Hz, Ar—H), 8.29 (1*H*, s, pyrazol-5-yl-H), 8.61 (1*H*, s, pyrazol-3-yl-H). ^13^C-NMR (CDCl_3_) δ 24.72, 54.56, 55.61, 114.67, 119.33, 121.15, 126.19, 127.75, 128.32, 140.30, 148.22, 158.91, 179.38. ν_max_ 3022, 2970, 2927, 1606, 1508, 1255.

### Refinement

H atoms bonded to the C atoms were fixed geometrically and treated as riding with C—H = 0.93 Å (aromatic) and 0.96 Å (methyl), with *U*_iso_(H) = 1.2 times those of the parent atoms for the aromatic H atoms and *U*_iso_(H) = 1.5 times those of the parent atoms for the methyl H atoms.

### Crystal data

**Table:**

C_20_H_18_ClN_3_O	*F*(000) = 736
*M**_r_* = 351.82	*D*_x_ = 1.382 Mg m^−^^3^
Monoclinic, *P*2_1_/*n*	Melting point = 464–465 K
Hall symbol: -P 2yn	Mo *K*α radiation, λ = 0.71073 Å
*a* = 11.635 (3) Å	Cell parameters from 858 reflections
*b* = 10.328 (3) Å	θ = 2.9–26.0°
*c* = 14.141 (4) Å	µ = 0.24 mm^−^^1^
β = 95.681 (5)°	*T* = 100 K
*V* = 1690.9 (8) Å^3^	Irregular, yellow
*Z* = 4	0.50 × 0.40 × 0.20 mm

### Data collection

**Table:**

Bruker SMART APEX CCD area-detector diffractometer	2662 reflections with *I* > 2σ(*I*)
Radiation source: fine-focus sealed tube	*R*_int_ = 0.092
graphite	θ_max_ = 26.4°, θ_min_ = 2.2°
phi and ω scans	*h* = −14→14
9553 measured reflections	*k* = −12→12
3461 independent reflections	*l* = −10→17

### Refinement

**Table:**

Refinement on *F*^2^	Primary atom site location: structure-invariant direct methods
Least-squares matrix: full	Secondary atom site location: difference Fourier map
*R*[*F*^2^ > 2σ(*F*^2^)] = 0.049	Hydrogen site location: inferred from neighbouring sites
*wR*(*F*^2^) = 0.124	H-atom parameters constrained
*S* = 0.97	*w* = 1/[σ^2^(*F*_o_^2^) + (0.0596*P*)^2^] where *P* = (*F*_o_^2^ + 2*F*_c_^2^)/3
3461 reflections	(Δ/σ)_max_ = 0.001
229 parameters	Δρ_max_ = 0.70 e Å^−^^3^
0 restraints	Δρ_min_ = −0.30 e Å^−^^3^

### Special details

**Table:**

Geometry. All e.s.d.'s (except the e.s.d. in the dihedral angle between two l.s. planes) are estimated using the full covariance matrix. The cell e.s.d.'s are taken into account individually in the estimation of e.s.d.'s in distances, angles and torsion angles; correlations between e.s.d.'s in cell parameters are only used when they are defined by crystal symmetry. An approximate (isotropic) treatment of cell e.s.d.'s is used for estimating e.s.d.'s involving l.s. planes.
Refinement. Refinement of *F*^2^ against ALL reflections. The weighted *R*-factor *wR* and goodness of fit *S* are based on *F*^2^, conventional *R*-factors *R* are based on *F*, with *F* set to zero for negative *F*^2^. The threshold expression of *F*^2^ > σ(*F*^2^) is used only for calculating *R*-factors(gt) *etc*. and is not relevant to the choice of reflections for refinement. *R*-factors based on *F*^2^ are statistically about twice as large as those based on *F*, and *R*- factors based on ALL data will be even larger.

### Fractional atomic coordinates and isotropic or equivalent isotropic displacement parameters (Å^2^)

**Table:**

	*x*	*y*	*z*	*U*_iso_*/*U*_eq_	
Cl1	1.22881 (4)	0.17525 (5)	0.17785 (4)	0.02511 (18)	
O1	0.48712 (12)	0.88812 (14)	0.58360 (11)	0.0251 (4)	
N1	1.03001 (14)	0.27790 (17)	0.29514 (12)	0.0184 (4)	
N2	0.81439 (14)	0.57762 (16)	0.43402 (12)	0.0190 (4)	
N3	0.91804 (14)	0.62821 (17)	0.41446 (12)	0.0203 (4)	
C1	1.04052 (17)	0.1445 (2)	0.27506 (14)	0.0180 (4)	
C2	1.12290 (17)	0.0837 (2)	0.22603 (14)	0.0208 (5)	
C3	1.11990 (18)	−0.0494 (2)	0.21418 (15)	0.0236 (5)	
H3	1.1750	−0.0904	0.1814	0.028*	
C4	1.03402 (18)	−0.1217 (2)	0.25156 (15)	0.0249 (5)	
H4	1.0329	−0.2112	0.2442	0.030*	
C5	0.95010 (19)	−0.0621 (2)	0.29962 (15)	0.0229 (5)	
H5	0.8922	−0.1105	0.3237	0.028*	
C6	0.95434 (17)	0.0709 (2)	0.31094 (14)	0.0192 (5)	
C7	0.87609 (17)	0.16310 (19)	0.35671 (14)	0.0182 (5)	
C8	0.93947 (17)	0.2890 (2)	0.34114 (14)	0.0171 (4)	
C9	0.86942 (19)	0.1327 (2)	0.46245 (15)	0.0254 (5)	
H9A	0.8386	0.0473	0.4688	0.038*	
H9B	0.8201	0.1947	0.4890	0.038*	
H9C	0.9454	0.1373	0.4957	0.038*	
C10	0.75543 (18)	0.1630 (2)	0.30166 (16)	0.0265 (5)	
H10A	0.7609	0.1928	0.2380	0.040*	
H10B	0.7054	0.2194	0.3327	0.040*	
H10C	0.7246	0.0767	0.3000	0.040*	
C11	0.90599 (17)	0.41488 (19)	0.37521 (14)	0.0183 (5)	
C12	0.80538 (18)	0.4512 (2)	0.41173 (14)	0.0205 (5)	
H12	0.7426	0.3980	0.4196	0.025*	
C13	0.97206 (17)	0.5295 (2)	0.37985 (14)	0.0196 (5)	
H13	1.0460	0.5349	0.3605	0.024*	
C14	0.73117 (18)	0.6587 (2)	0.47155 (14)	0.0189 (5)	
C15	0.76750 (18)	0.7680 (2)	0.52286 (14)	0.0209 (5)	
H15	0.8458	0.7879	0.5318	0.025*	
C16	0.68888 (18)	0.8471 (2)	0.56077 (15)	0.0210 (5)	
H16	0.7138	0.9209	0.5946	0.025*	
C17	0.57208 (18)	0.8165 (2)	0.54834 (15)	0.0202 (5)	
C18	0.53577 (18)	0.7079 (2)	0.49591 (15)	0.0223 (5)	
H18	0.4575	0.6879	0.4869	0.027*	
C19	0.61435 (18)	0.6295 (2)	0.45722 (15)	0.0215 (5)	
H19	0.5894	0.5573	0.4216	0.026*	
C20	0.5233 (2)	1.0001 (2)	0.63873 (16)	0.0274 (5)	
H20A	0.5720	0.9738	0.6942	0.041*	
H20B	0.4567	1.0442	0.6577	0.041*	
H20C	0.5654	1.0572	0.6012	0.041*	

### Atomic displacement parameters (Å^2^)

**Table:**

	*U*^11^	*U*^22^	*U*^33^	*U*^12^	*U*^13^	*U*^23^
Cl1	0.0224 (3)	0.0279 (3)	0.0255 (3)	0.0004 (2)	0.0048 (2)	0.0008 (2)
O1	0.0289 (8)	0.0239 (8)	0.0229 (9)	0.0037 (7)	0.0038 (7)	−0.0050 (7)
N1	0.0208 (9)	0.0183 (9)	0.0151 (9)	0.0010 (7)	−0.0019 (7)	0.0004 (7)
N2	0.0212 (9)	0.0192 (9)	0.0161 (9)	−0.0004 (7)	−0.0009 (7)	0.0000 (7)
N3	0.0212 (9)	0.0220 (9)	0.0174 (9)	−0.0015 (8)	0.0007 (8)	0.0005 (7)
C1	0.0186 (10)	0.0201 (11)	0.0142 (11)	0.0020 (8)	−0.0041 (9)	0.0015 (8)
C2	0.0188 (10)	0.0269 (12)	0.0161 (11)	−0.0005 (9)	−0.0014 (9)	0.0028 (9)
C3	0.0260 (11)	0.0249 (12)	0.0193 (12)	0.0062 (10)	−0.0011 (9)	−0.0027 (9)
C4	0.0281 (12)	0.0207 (11)	0.0249 (13)	0.0018 (10)	−0.0031 (10)	−0.0016 (9)
C5	0.0244 (11)	0.0234 (12)	0.0204 (12)	−0.0033 (9)	−0.0003 (9)	−0.0007 (9)
C6	0.0221 (10)	0.0214 (11)	0.0128 (11)	0.0003 (9)	−0.0038 (8)	0.0014 (8)
C7	0.0203 (10)	0.0193 (11)	0.0147 (11)	0.0002 (8)	0.0008 (9)	0.0001 (8)
C8	0.0195 (10)	0.0204 (11)	0.0103 (10)	0.0015 (8)	−0.0046 (8)	0.0017 (8)
C9	0.0333 (12)	0.0233 (11)	0.0203 (12)	0.0002 (10)	0.0059 (10)	0.0018 (9)
C10	0.0231 (11)	0.0272 (12)	0.0287 (13)	0.0003 (10)	−0.0002 (10)	−0.0067 (10)
C11	0.0215 (10)	0.0200 (11)	0.0127 (10)	0.0017 (9)	−0.0017 (8)	0.0038 (8)
C12	0.0244 (11)	0.0178 (11)	0.0188 (11)	−0.0020 (9)	−0.0009 (9)	0.0013 (8)
C13	0.0190 (10)	0.0234 (11)	0.0160 (11)	0.0028 (9)	−0.0002 (9)	0.0021 (9)
C14	0.0232 (11)	0.0202 (11)	0.0127 (11)	0.0035 (9)	−0.0007 (9)	0.0017 (8)
C15	0.0222 (10)	0.0231 (11)	0.0162 (11)	−0.0006 (9)	−0.0035 (9)	0.0027 (9)
C16	0.0275 (11)	0.0183 (11)	0.0163 (11)	−0.0019 (9)	−0.0020 (9)	−0.0008 (9)
C17	0.0269 (11)	0.0200 (11)	0.0133 (11)	0.0025 (9)	0.0008 (9)	0.0023 (8)
C18	0.0207 (10)	0.0249 (12)	0.0206 (12)	−0.0013 (9)	−0.0010 (9)	0.0005 (9)
C19	0.0274 (11)	0.0182 (11)	0.0179 (11)	−0.0022 (9)	−0.0020 (9)	−0.0023 (9)
C20	0.0393 (14)	0.0223 (12)	0.0206 (12)	0.0051 (10)	0.0030 (10)	−0.0042 (9)

### Geometric parameters (Å, °)

**Table:**

Cl1—C2	1.744 (2)	C9—H9A	0.9600
O1—C17	1.367 (2)	C9—H9B	0.9600
O1—C20	1.434 (2)	C9—H9C	0.9600
N1—C8	1.297 (2)	C10—H10A	0.9600
N1—C1	1.415 (3)	C10—H10B	0.9600
N2—C12	1.344 (3)	C10—H10C	0.9600
N2—N3	1.367 (2)	C11—C12	1.378 (3)
N2—C14	1.422 (3)	C11—C13	1.410 (3)
N3—C13	1.317 (3)	C12—H12	0.9300
C1—C2	1.387 (3)	C13—H13	0.9300
C1—C6	1.393 (3)	C14—C15	1.384 (3)
C2—C3	1.385 (3)	C14—C19	1.387 (3)
C3—C4	1.393 (3)	C15—C16	1.375 (3)
C3—H3	0.9300	C15—H15	0.9300
C4—C5	1.388 (3)	C16—C17	1.389 (3)
C4—H4	0.9300	C16—H16	0.9300
C5—C6	1.383 (3)	C17—C18	1.388 (3)
C5—H5	0.9300	C18—C19	1.375 (3)
C6—C7	1.507 (3)	C18—H18	0.9300
C7—C8	1.521 (3)	C19—H19	0.9300
C7—C10	1.536 (3)	C20—H20A	0.9600
C7—C9	1.537 (3)	C20—H20B	0.9600
C8—C11	1.453 (3)	C20—H20C	0.9600
			
C17—O1—C20	116.76 (16)	C7—C10—H10A	109.5
C8—N1—C1	106.09 (17)	C7—C10—H10B	109.5
C12—N2—N3	111.93 (16)	H10A—C10—H10B	109.5
C12—N2—C14	128.26 (17)	C7—C10—H10C	109.5
N3—N2—C14	119.80 (16)	H10A—C10—H10C	109.5
C13—N3—N2	104.03 (16)	H10B—C10—H10C	109.5
C2—C1—C6	119.53 (19)	C12—C11—C13	103.48 (18)
C2—C1—N1	128.23 (18)	C12—C11—C8	129.33 (19)
C6—C1—N1	112.24 (17)	C13—C11—C8	127.18 (18)
C3—C2—C1	119.95 (19)	N2—C12—C11	107.65 (18)
C3—C2—Cl1	120.07 (16)	N2—C12—H12	126.2
C1—C2—Cl1	119.97 (17)	C11—C12—H12	126.2
C2—C3—C4	119.77 (19)	N3—C13—C11	112.91 (18)
C2—C3—H3	120.1	N3—C13—H13	123.5
C4—C3—H3	120.1	C11—C13—H13	123.5
C5—C4—C3	120.9 (2)	C15—C14—C19	119.85 (19)
C5—C4—H4	119.5	C15—C14—N2	119.45 (18)
C3—C4—H4	119.5	C19—C14—N2	120.70 (18)
C6—C5—C4	118.58 (19)	C16—C15—C14	120.6 (2)
C6—C5—H5	120.7	C16—C15—H15	119.7
C4—C5—H5	120.7	C14—C15—H15	119.7
C5—C6—C1	121.21 (19)	C15—C16—C17	119.71 (19)
C5—C6—C7	131.37 (19)	C15—C16—H16	120.1
C1—C6—C7	107.40 (17)	C17—C16—H16	120.1
C6—C7—C8	98.95 (16)	O1—C17—C18	116.06 (18)
C6—C7—C10	110.03 (17)	O1—C17—C16	124.34 (19)
C8—C7—C10	111.04 (17)	C18—C17—C16	119.59 (19)
C6—C7—C9	112.32 (17)	C19—C18—C17	120.63 (19)
C8—C7—C9	112.76 (17)	C19—C18—H18	119.7
C10—C7—C9	111.17 (17)	C17—C18—H18	119.7
N1—C8—C11	120.19 (19)	C18—C19—C14	119.63 (19)
N1—C8—C7	115.26 (18)	C18—C19—H19	120.2
C11—C8—C7	124.54 (18)	C14—C19—H19	120.2
C7—C9—H9A	109.5	O1—C20—H20A	109.5
C7—C9—H9B	109.5	O1—C20—H20B	109.5
H9A—C9—H9B	109.5	H20A—C20—H20B	109.5
C7—C9—H9C	109.5	O1—C20—H20C	109.5
H9A—C9—H9C	109.5	H20A—C20—H20C	109.5
H9B—C9—H9C	109.5	H20B—C20—H20C	109.5
			
C12—N2—N3—C13	−0.1 (2)	C10—C7—C8—C11	67.3 (2)
C14—N2—N3—C13	−178.87 (18)	C9—C7—C8—C11	−58.2 (3)
C8—N1—C1—C2	179.1 (2)	N1—C8—C11—C12	168.9 (2)
C8—N1—C1—C6	−0.8 (2)	C7—C8—C11—C12	−12.2 (3)
C6—C1—C2—C3	−0.8 (3)	N1—C8—C11—C13	−12.4 (3)
N1—C1—C2—C3	179.3 (2)	C7—C8—C11—C13	166.5 (2)
C6—C1—C2—Cl1	178.18 (15)	N3—N2—C12—C11	−0.4 (2)
N1—C1—C2—Cl1	−1.6 (3)	C14—N2—C12—C11	178.24 (19)
C1—C2—C3—C4	0.0 (3)	C13—C11—C12—N2	0.7 (2)
Cl1—C2—C3—C4	−179.00 (16)	C8—C11—C12—N2	179.67 (19)
C2—C3—C4—C5	0.9 (3)	N2—N3—C13—C11	0.6 (2)
C3—C4—C5—C6	−0.9 (3)	C12—C11—C13—N3	−0.8 (2)
C4—C5—C6—C1	0.1 (3)	C8—C11—C13—N3	−179.82 (19)
C4—C5—C6—C7	178.4 (2)	C12—N2—C14—C15	155.5 (2)
C2—C1—C6—C5	0.8 (3)	N3—N2—C14—C15	−26.0 (3)
N1—C1—C6—C5	−179.35 (19)	C12—N2—C14—C19	−24.5 (3)
C2—C1—C6—C7	−177.89 (18)	N3—N2—C14—C19	154.00 (19)
N1—C1—C6—C7	2.0 (2)	C19—C14—C15—C16	0.8 (3)
C5—C6—C7—C8	179.4 (2)	N2—C14—C15—C16	−179.27 (18)
C1—C6—C7—C8	−2.1 (2)	C14—C15—C16—C17	0.7 (3)
C5—C6—C7—C10	−64.3 (3)	C20—O1—C17—C18	179.43 (18)
C1—C6—C7—C10	114.23 (19)	C20—O1—C17—C16	−1.5 (3)
C5—C6—C7—C9	60.1 (3)	C15—C16—C17—O1	179.54 (19)
C1—C6—C7—C9	−121.36 (19)	C15—C16—C17—C18	−1.5 (3)
C1—N1—C8—C11	178.18 (17)	O1—C17—C18—C19	179.89 (18)
C1—N1—C8—C7	−0.8 (2)	C16—C17—C18—C19	0.8 (3)
C6—C7—C8—N1	1.9 (2)	C17—C18—C19—C14	0.6 (3)
C10—C7—C8—N1	−113.7 (2)	C15—C14—C19—C18	−1.4 (3)
C9—C7—C8—N1	120.8 (2)	N2—C14—C19—C18	178.62 (19)
C6—C7—C8—C11	−177.07 (18)		
